# Navigating change: the trajectory of the Jornal Brasileiro de Pneumologia in a dynamic scientific landscape and its international recognition

**DOI:** 10.36416/1806-3756/e20250331

**Published:** 2025-11-19

**Authors:** Bruno Guedes Baldi, Rogério Souza

**Affiliations:** 1. Divisao de Pneumologia, Instituto do Coracao - InCor - Hospital das Clinicas - HCFMUSP - Faculdade de Medicina, Universidade de São Paulo, São Paulo (SP) Brasil.; 2. Hospital do Coração, São Paulo (SP) Brasil.

Over the fifty years of the foundation of the *Jornal Brasileiro de Pneumologia* (JBP), initially named *Jornal de Pneumologia*, it has evolved from a national to an international scope, especially in the last two decades. The JBP followed the continuous transformation of science and adapted itself to emerging trends, shifting paradigms and evolving methodologies in the research of respiratory diseases. The main objectives of the JBP are publishing changes and highlights in respiratory medicine, as well as to act as a source of dissemination of the Brazilian and international research in respiratory medicine and correlated areas, with a progressive increase in its scientific rigor. Additionally, the Journal has an important role in improving clinical care on respiratory health internationally, with a particular focus on Brazil and other countries in Latin America.

The JBP is currently the major Latin American journal in the respiratory medicine field and a continuous and robust source of knowledge and support in this region. Several demands are covered by the JBP, such as the publication of guidelines primarily addressing diagnosis and treatment, and epidemiological and daily life studies that help clinical practice, including some diseases prevalent in our environment, such as tuberculosis and other infectious diseases.^1^ In addition, the studies published in the Journal helped the implementation of public health policies and the approval of prescribing high-cost medications by regulatory agencies in Brazil.^2^ ^,^ ^3^

It is pivotal to highlight not only the national impact of the JBP, but also the progressive increase in its international recognition, currently the Journal being placed among the major Brazilian medical journals and at a significant position within the respiratory medicine journals in the major international indexes agencies, such as Scimago Journal & Country Rank (SJR) and Journal Citation Reports (JCR).^4^ ^,^ ^5^ Several achievements were obtained by the JBP in its history towards greater visibility, recognition, and internationalization. The journal was included in the SciELO database in 2002 and indexed in PubMed/MEDLINE, which is sponsored by the United States National Library of Medicine, in 2006, making the Journal accessible for foreign readers and increasing the visibility and citation potential of its articles.^6^ The Journal achieved other milestones when it was indexed in the Institute for Scientific Information (ISI) Web of Knowledge in 2009, and when its first impact factor was published in the JCR in 2012.^7^ In recent years, there has been a progressive increase in impact factor of the JBP in the main databases, culminating at a score of 3.0 in the JCR 2024, published this year, reinforcing its scientific excellence and its international recognition in the respiratory medicine.^4^ The combined role of authors, reviewers, and editorial board members, with the expansion of representatives from other countries, in addition to the support of the staff and board of directors of the Brazilian Thoracic Society, was fundamental to the growth and progressive increase of the international relevance of the JBP.

Several important manuscripts have been published and received a relevant number of citations in the JBP in recent years, such as those related to tuberculosis and other mycobacteria, COVID-19, smoking, obstructive diseases, among others. It is also essential to emphasize the adaptations that the JBP had to make in order to face the challenges determined by COVID-19. The Journal received an increasing number of COVID-19-related submissions and was required to determine those that would be approved carefully, without forgetting the importance to continue publishing manuscripts on other topics.^8^ ^-^ ^10^ Then, it was necessary to make the editorial processes faster during the pandemics, without losing scientific rigor.^10^

It is also important to highlight the increasing number of guidelines, review articles, and meta-analyses published in the JBP, reinforcing its commitment to updating topics for the practice of pulmonologists and other related professionals. The number of articles published by international authors, including editorials, has also progressively increased over the years.^11^ Another aspect that reinforces the greater internationalization of the JBP is the consolidated partnership with other relevant journals in respiratory medicine, such as the Pulmonology Journal and the *Archivos de Bronconeumologia*. Other factors that contributed to the greater visibility and submission of articles to the JBP include its the open access, as well as the submission and publication of articles free of charge and only in English, the increase in social media dissemination of information, and the continuous publication of manuscripts.

In summary, several transformations have been implemented in the JBP since its first release in 1975, leading to its consolidation as a relevant journal in the field of respiratory diseases and an important source of reference for pulmonologists in Brazil and abroad. In this context, the Journal has highlighted pivotal contributions that helped to shape the knowledge and our understanding of lung diseases over the years. It is hoped that the JBP will increasingly inspire future generations of researchers, pulmonologists, and other health professionals over the next 50 years, with a commitment to achieve a progressively higher quality and maintaining scientific rigor without forgetting to address important topics for the daily practice of respiratory care professionals.
